# Chitosan-Coated Halloysite Nanotubes As Vehicle for Controlled Drug Delivery to MCF-7 Cancer Cells In Vitro

**DOI:** 10.3390/ma14112837

**Published:** 2021-05-26

**Authors:** Emmanuel Nyankson, Shadrack O. Aboagye, Johnson Kwame Efavi, Benjamin Agyei-Tuffour, Lily Paemka, Bernard O. Asimeng, Srinivasan Balapangu, Patrick K. Arthur, Elvis K. Tiburu

**Affiliations:** 1 Department of Materials Science and Engineering, University of Ghana, P.O. Box LG77 Legon, Ghana; enyankson@ug.edu.gh (E.N.); shadrackaboagye@gmail.com (S.O.A.); jkefavi@ug.edu.gh (J.K.E.); bagyei-tuffour@ug.edu.gh (B.A.-T.); 2 Department Biochemistry, Cell and Molecular Biology, University of Ghana, P.O. Box LG54 Legon, Ghana; LPaemka@ug.edu.gh (L.P.); parthur14@gmail.com (P.K.A.); 3 West Africa Center for Cell Biology of Infectious Pathogens, University of Ghana, Legon, Ghana; srinivasan_bs85@yahoo.com; 4 Department of Biomedical Engineering, University of Ghana, P.O. Box LG77 Legon, Ghana; boasimeng@ug.edu.gh

**Keywords:** curcumin, halloysite nanotubes, chitosan, breast cancer cell line, sustained and targeted release

## Abstract

The aim of the work is to improve the release properties of curcumin onto human breast cancer cell lines using coated halloysite nanotubes (HNTs) with chitosan as a polycation. A loading efficiency of 70.2% (*w*/*w*) was attained for loading 4.9 mg of the drug into 0.204 g bed volume of HNTs using the vacuum suction method. Results acquired from Brunauer-Emmett-Teller (BET), Fourier-transform infrared (FTIR) spectroscopy, transmission electron microscopy (TEM), scanning electron spectroscopy (SEM), zeta potential, and thermogravimetric analysis (TGA) indicated the presence of the drug and the biopolymer in and around the nanotubes. The release properties of drug-loaded HNTs (DLHNTs) and chitosan-coated drug-loaded HNTs (DLHNTs-CH) were evaluated. The release percentages of DLHNTs and DLHNTs-CH after 6 h were 50.7 and 37%, respectively. Based on the correlation coefficients obtained by fitting the release nature of curcumin from the two samples, the Korsmeyer-Peppas model was found to be the best-fitted model. In vitro cell viability studies were carried out on the human breast cancer cell line MCF-7, using the MTT and trypan blue exclusion assays. Prior to the Trypan blue assay, the IC_50_ of curcumin was determined to be ~30 µM. After 24 h of incubation, the recorded cell viability values were 94, 68, 57, and 51% for HNTs, DLHNTs-CH, DLHNTs, and curcumin, respectively. In comparison to the release studies, it could be deducted that sustained lethal doses of curcumin were released from the DLHNTs-CH within the same time. It is concluded from this work that the “burst release” of naked drugs could be slowly administered using chitosan-coated HNTs as potential drug carriers.

## 1. Introduction

There has been a study rise in number of new cancer cases globally. The cancer cases were reported to be 14.1 and 18.1 million in 2012 and 2018, respectively; however, this value is estimated to increase to 29.4 million by 2040. The number of deaths was reported to be 8.1 and 10 million in 2012 and 2020, respectively [1,2]. Breast cancer is known to be the most predominant disease amongst all possible malignancies that affect women [3]. One in eight women are diagnosed annually, which translates to 2900 new cases, making breast cancer one of the leading causes of death especially in sub-Sahara Africa (sSA) [4]. In spite of the high mortality rate outlined, patients with early-stage breast cancer have high chances of survival, as they can be treated with surgery, radiation and chemotherapy. However, if the cancer metastasizes, then oncologists need to administer drugs systemically to reach and eliminate the cancer cells [5]. Inconsistency in chemotherapy dosages however makes metastatic breast cancer a pertinent problem [6]. It has been documented that encapsulating anticancer drugs in nanoparticles can reduce the toxicity of the active substances significantly, thereby achieving optimum success [7]. However, if “burst release”, which is a common phenomenon where an initial high dose of a delivered drug is made available to tissues and organs upon first contact, occurs, anti-cancer drugs tend to lose their efficacy due to degradation within a short period of time [8,9]. There is therefore a need to curtail the limitation created by “burst release” by employing the principle of controlled/sustained release to improve the efficacy of anticancer agents [10].

A polyphenol, curcumin (diferuloylmethane), isolated from the root of the *Curcuma longa* plant, has been used over the years for decoration, food preservation, as well as for medicinal purposes [11]. Curcumin has been shown to act as an anti-atherosclerotic agent, amplify bile secretion, eliminate fibrosis, protect against cataract formation, and downregulate growth factor receptors such as HER-2 [11]. Despite the aforementioned characteristics, similar to other anti-cancer drugs, curcumin has several limitations such as easy degradation and low bioavailability [12] and must be encapsulated to enhance its efficacy towards its target site [13].

Nanocomposites when used as drug delivery systems are able to penetrate tumor micro-environments and slowly release loaded drugs for sustainable periods [10,14]. Over the years, several clay minerals such as montmorillonite (MMT) [15], zeolite [16], bentonite [17], and silica [18,19] have been used in creating drug transport devices. However, due to several reported limitations such as poor drug releasing efficiency, hydrophobicity, excessive cytotoxicity, and poor clearance rate from blood [20], these materials tend not to be suitable for biomedical applications.

Halloysite nanotubes (HNTs) are aluminosilicate minerals formed when flat sheets of kaolin naturally roll into empty tubes with diameters of ca. 50 nm and length of ca. 1 μm [9]. The stoichiometry of halloysite is Al_2_Si_2_O_5_(OH)_4_·nH_2_O and occurs in two forms, the hydrated form with enlarged interlayer spacing of ca. 10 Å [21] and the anhydrous form with a reduced interlayer spacing of ~7 Å. Integration of water in the inter-lamellar spaces causes the enlargement in the interlayer spacing [22,23]. Distinguished properties such as high surface area and ability to interact with many active agents through surface adsorption or ion exchange response, coupled with their biocompatibility, makes HNTs a successful candidate for a drug delivery vehicle [23] and other environmental applications [24,25,26,27,28]. Since the surface of an HNT is negatively charged, polycations such as chitosan can be coated onto HNTs. By employing a coated approach using biopolymers, the release of drugs loaded into the lumen of HNTs may be sustained over a long period of time. Additionally, ligands can be attached to the surface of HNTs based solely on electrochemical interactions for active targeting of tumors [29,30]. Tumor residence time has been suggested to improve significantly by biomimetic composite materials such as chitosan-gelatin polymer networks, which enhance initial cell adhesion [31].

In this work, curcumin was entrapped into the lumen of halloysite with the aid of vacuum suction and release. Thereafter, the drug-loaded halloysite was coated with chitosan. The uncoated and coated HNTs were characterized by FTIR, BET, TEM, TGA, SEM, and zeta potential measurements. The release rate and kinetics of the coated and uncoated HNTs were investigated using existing kinetic models. Cell viability (CV) studies were carried out using the MTT and Trypan Blue exclusive assays.

## 2. Materials and Methods

### 2.1. Reagents and Materials

Curcumin (99% purity), halloysite nanotubes Al_2_Si_2_O_5_(OH)_4_·2H_2_O (99.5% purity), Trypan Blue Solution (0.4% in phosphate buffered saline (PBS)), medium molecular weight chitosan (Mw 200,000 Da), dimethyl sulfoxide (DMSO), fetal bovine serum (FBS), Dulbecco’s modified Eagle’s Medium (DMEM), penicillin/streptomycin, trypsin thiazolyl blue tetrazolium bromide, and phosphate buffered saline were purchased from Sigma-Aldrich (Gillingham, UK).

### 2.2. Drug Loading

Approximately 5 mg of curcumin was loaded/entrapped into 0.2 g bed volume of HNTs via the procedure described by Price et al. [21]. A volume of 5 mL of absolute ethanol was used to dissolve approximately 25 mg of dry curcumin powder to attain a stock solution at a concentration of 5 mg/mL. A volume of 5 μL of the stock solution was diluted to obtain a working solution of 5 μg/mL for UV-Vis analysis. Two hundred milligrams (200 mg) of HNTs was then dispersed in 1 mL of 5 mg/mL curcumin solution. The resultant mixture was sonicated for about 30 min to ensure uniform mixing and then introduced into a vacuum chamber. Multiple vacuum suction and release cycles at conventional time intervals were performed until the fizzing stopped. Dry powder was obtained by drying the resulting mixture in a vacuum for 3 h. The unbound drug was washed by centrifuging the loaded HNTs at 6000 rpm for 20 min. The supernatant was analyzed with UV-Vis at an absorbance wavelength of 420 nm. The washing was repeated several times to ensure all residual curcumin had been removed. The drug-loaded HNTs designated as DLHNTs were transferred into a 1.5 mL Eppendorf tube.

The loading efficiency of curcumin and the percent entrapment onto HNTs was evaluated using the Shi et al. equations [32]. Equations (1) and (2) were used to evaluate the loading efficiency and the loading percentage, respectively.

(1) $$\mathrm{Loading}\;\mathrm{efficiency}\;(\mathrm{LE})\;(\%)=\frac{M_{t}}{M_{0}}\times 100\%$$

M_t_ refers to the mass of the loaded drug, while Mo refers to the total mass of the drug used for loading [32].

(2) $$\mathrm{Loading}\;\mathrm{Percentage}\;(\mathrm{LP})\;(\%)=\frac{M_{t}}{M}\times 100\%$$

M_t_ refers to the mass of the loaded drug, while M refers to the total mass of the loaded vehicle [32].

The drug loading experiment was conducted in triplicate and the average with standard deviations was reported.

### 2.3. Nitrogen Adsorption of Drug-Loaded Halloysite Nanotubes (DLHNTs)

The surface area of mesoporous materials can be evaluated via the Brunauer-Emmet-Teller (BET) equation presented below (Equation (3)) for relative pressures between 0.05 and 0.3 (P/PO = 0.05–0.3) and was carried out at a constant temperature of 77 K.

(3) $$\frac{P}{V_{\alpha \left(P_{0}-P\right)}}=\frac{1}{V_{m}C}+\frac{C-1}{V_{m}C}\;\left(\frac{P}{P_{0}}\right)\;$$

where *p* = pressure, $P_{0}$ = saturation pressure, *V_a_* = volume adsorbed, *V_m_* = monolayer volume, and *C* = multilayer adsorption parameter.

Using the Novawin v11.0 (Boynton Beach, FL, USA) analysis software on the Micromeritics ASAP 2020 instrument (Micrometrics, Norcross, GA, USA), porosity and surface area of the materials were measured via N_2_ adsorption. Prior to analysis, the samples were degassed below vacuum at 120 °C for 24 h. The adsorption and desorption isotherms were recorded at approximately −196 °C.

### 2.4. Coated Halloysite Nanotubes (HNTs) Using Chitosan

A traditional technique was employed to coat Chitosan (polycation) on the surface of drug-loaded HNTs (DLHNTs) [33]. Three milligrams of chitosan powder in 1 mL of 2% acetic acid was prepared and 1 mL of the chitosan solution introduced into 100 mg of DLHNTs. The suspension was then gently stirred in an incubator at 37 °C for 1 h. Excess polyelectrolytes were washed off by centrifuging the resultant solution at 6000 rpm for 5 min. After centrifugation, the sample was dried in a vacuum at room temperature. The coated DLHNTs with chitosan were designated as DLHNTs-CH.

### 2.5. Characterization

#### 2.5.1. Thermogravimetric Analysis (TGA) of HNTs, DLHNTs and DLHNTs-CH

TGA was carried out on the samples to evaluate their respective mass losses when subjected to successive heating. Thermogravimetric analysis was done using TGA Instrument Q500 V 6.7 (TA Instruments, New Castle, DE, USA) below flowing N_2_/O_2_ (60:40, *v*/*v*, mL/min). The heating was done from 25 to 800 °C at a heating rate of 10 °C/min.

#### 2.5.2. Fourier Transform Infrared Spectroscopy (FTIR) of HNTs, DLHNTs, and DLHNTs-CH

The Fourier-transformed infrared spectra were acquired by the usage of a Thermo Nicolet 50 spectrophotometer equipped with a diamond Attenuated Total Reflection (ATR) crystal. The analysis was done from 500 to 4000 cm^−1^ and the data was analyzed employing OMNIC 9.2.98 (Thermo Fisher Scientific, Gloucester, UK).

#### 2.5.3. Scanning Electron Microscopy (SEM) Analysis of HNTs, DLHNTs, and DLHNTs-CH

The FEI Nova NanoSem (Thermo Fisher Scientific, Gloucester, UK) scanning electron microscope was used to examine the morphology of the samples. Before the analysis, the samples were affixed with the aid of carbon tape onto a stainless steel stub and were sputter coated with a thin layer of Pt. The images were obtained using a probe at 3 mA and an accelerating voltage of 15 kV.

#### 2.5.4. Transmission Electron Microscope (TEM) of HNTs, DLHNTs, and DLHNTs-CH

By using the aberration-corrected JEOL TEM (model JEM-1400, JEOL, Zurich, Switzerland) at 200 kV and analysis software ‘Gatan Digital Micrograph’, high-resolution high-angle annular darkish field TEM images were obtained for all the samples. The samples were drop cast on a 200-mesh carbon covered copper grid after they were dispersed in methanol and dried thereafter in ambient conditions.

#### 2.5.5. Zeta Potential Analysis of HNTs and DLHNTs-CH

The analysis was done using Zetasizer Ver. 7.10 (Malvern Panalytical Company, Malvern, UK), at a temperature of 25 °C, and 12 zeta runs were undertaken with the dispersant being distilled water. Data were analyzed using Zeta Potential Overlay Report ver. 2.1 (Malvern Panalytical Company, Malvern, UK).

### 2.6. In Vitro Curcumin Release from DLHNTs and DLHNTs-CH

Fifty milligrams of the curcumin-loaded halloysite (DLHNTs) was weighed and dispersed in a 100 mL release medium made up of an ethanol-phosphate buffer solution (*v*/*v*, 1:1) at pH 6.3 under steady stirring at 50 rpm. At fixed time intervals, 1 mL of the sample was pipetted and centrifuged at 6000 rpm for 20 min. A sequence of ten samples were collected for UV-Vis analysis. Absorbance for each sample was measured at a maximum wavelength of 420 nm. The percentage of the released drug at each time interval was evaluated using Equation (4). Based on the average results obtained by conducting the studies in triplicate, the release curves were plotted.

(4) $$\mathrm{Release}\;(\%)=\frac{\mathrm{Amount}\;\mathrm{of}\;\mathrm{drug}\;\mathrm{released}}{\mathrm{Total}\;\mathrm{amount}\;\mathrm{of}\;\mathrm{drug}-\mathrm{loaded}}\times 100$$

An MTT colorimetric assay was performed to examine the antitumor effect of curcumin. The MTT assay is based on the selective ability of either normal or cancerous cells to reduce the tetrazolium dye MTT 3-(4,5-dimethylthiazol-2-yl)-2,5-diphenyltetrazolium bromide (yellow soluble salt) to an insoluble formazan (purple-blue) precipitate. MCF-7 cells were seeded in triplicate at 10,000 cells/100 μL in 96-well plates overnight. Next, the wells were treated with different concentrations of curcumin in 1% DMSO, ranging from 0 to 150 μM and incubated overnight. At the end of the incubation period, 10 µL of the insoluble dye in PBS at a concentration of 1 mg/mL was added to each well. The dye was solubilized with 100 μL of acidified isopropanol, after which the plate was further incubated for 3 h. A Thermo Fisher Varioskan LUX Multimode Microplate (Thermo Fisher Scientific, Gloucester, UK) reader spectrophotometer was used to record the optical densities (changes in absorbance) at 570 nm after a one-hour incubation. The cytotoxicity of raw HNT, DLHNT, and DLHNT-CH was determined using this same approach. For this experiment, 15,000 cells/well were seeded in a 96-well plate for 24 h. Concentrations of HNTs (DLHNT, DLHNT-CH) at 50 to 1000 μg/mL were co-cultured with the cells after the incubation period.

The cell viability percentage was calculated according to the following Equations:

(5) $$\mathrm{Cytotoxicity}\;(\%)=1-\frac{Mean\;absorbance\;of\;inhibitor}{Mean\;absorbance\;of\;negative\;control}\times 100$$

Viability (%) = 100 − Cytotoxicity (%)
(6)

### 2.7. Trypan Blue Exclusion Assay Studies

When trypan blue dye is applied to cells, permeable cell membrane (non-viable) cells stain dark blue, while cells with intact plasma membranes remain unstained [34]. The treated cells were incubated for 24 h, after which they were collected by trypsinization and washed with PBS. The cells were then separated into different Eppendorf tubes per sample (1.5 mL) and resuspended in PBS (1 mL). Twenty micrograms of the cell suspension was pipetted and mixed with an equal volume of Trypan Blue (0.4% in PBS) *v*/*v* 1:1. After this was done, 20 µL of the resultant mixture was pipetted and introduced into the vacuum enclosure of a cellometer counting chamber. The counting chamber was slotted into the Nexcellon Cellometer cell counter and analyzed using the Nexcellon v11 analysis software (Nexcelom, Manchester, UK). All experiments were done in triplicate.

## 3. Results and Discussion

### 3.1. HNT Curcumin Loading and Entrapment

The loading efficiency of curcumin and the percent entrapment onto HNTs was evaluated. The loading efficiencies and percentage of the curcumin-loaded halloysite nanotubes was 70.2%, using ca. 5 mg of the drug and ca. 0.2 g of the HNTs suspension.

### 3.2. Nitrogen Adsorption of HNTs and DLDHNTs

**Figure 1:**
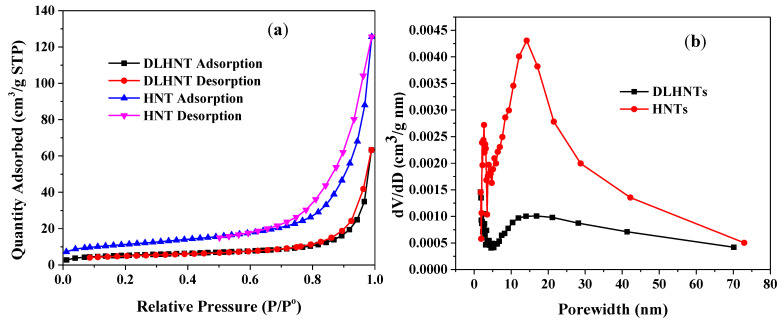
(**a**) The nitrogen adsorption and desorption isotherms of HNTs and DLHNTs, (**b**) BJH pore size distributions for HNTs and DLHNTs.

The Brunauer-Emmet-Teller (BET) analysis of the adsorption-desorption nitrogen isotherms was employed to confirm the loading of curcumin into the lumen of the halloysite nanotubes. As shown in Figure 1a, the adsorption-desorption isotherms for raw halloysite (HNTs) and drug-loaded halloysite nanotubes (DLHNTs) depict an IV isotherm with a H_3_ hysteresis loop. Slit-shaped pores are normally observed in materials that give rise to a H_3_ hysteresis loop. In comparison with the raw HNTs, the drug-loaded halloysite nanotubes exhibit narrowing of the absorption/desorption hysteresis. The low rates of absorbed/desorbed N_2_ indicated that the drug molecules were able to occupy and fill the pores of the HNT. The BET surface area and the total pore volume of the HNTs recorded higher values in comparison to the DLHNTs.

Figure 1b indicated the Barrett-Joyner-Halenda (BJH) pore size distributions (determined from the desorption isotherms) of the raw and drug-loaded halloysites. Owing to pore formation via agglomeration of the tubes into bundles, pores arising from the lumen, and intercalated areas (Figure 1b), the halloysites were characterized by a broad spectrum of pore sizes ranging from 2 to 75 nm and confirmed earlier studies by White et al. [35]. This is valid due to the vast distribution of the pores’ characteristics associated with HNTs [36]. The peak at around 2–3.5 nm pore diameter could be related to fine intra-tubular pores. A range of larger pores between ~6–75 nm (with a higher intensity at ~14 nm) was also found in the BJH pore size distribution in Figure 1b. The larger pores can be attributed to the lumen of HNTs. The change in the pore diameter after loading HNTs with the curcumin drug is evident from Figure 1b. The surface area and total pore volume were 38.95 m^2^/g and 0.11 cm^3^/g, and 18.21 m^2^/g and 0.04 cm^3^/g for HNTs and DLHNTs, respectively, as shown in Table 1.

### 3.3. Zeta Potential Measurements of HNTs and DLHNTs-CH

**Figure 2:**
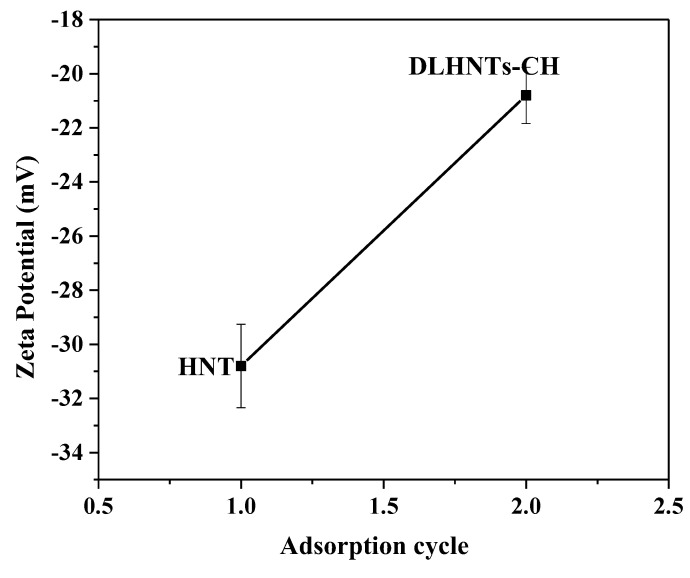
Zeta potential measurement of HNTs and drug-loaded HNTs coated with CH. Experiment was conducted in triplicate.

The surface charge on HNTs and DLHNTs was monitored using zeta potential measurements, as shown in Figure 2. The analysis was conducted using deionized water as a dispersant at neutral pH. Ideal dispersion and charging of HNTs in a wide variety of pH are possible due to the inner and outer hydroxyl groups, which act as major reactive sites [37]. It is apparent from Figure 2 that the surface charge of HNTs reaches an average value of approximately −30 mV. The high negative charge recorded on the surface could be ascribed to the difference in functional groups on the surface (silica) and the lumen (alumina) of the nanotubes [38]. The formation of the surface hydroxyl group resulted from exposing each oxide to water. The surface charge is mostly negative over a broad spectrum of pH, since the surface is basically composed of silica [39] as shown in Figure 2. Addition of the chitosan layer to HNTs shifted the negative polarity of the HNTs to a more positive region (approximately −20 mV) in water due to the positive attributes of the polycation.

### 3.4. Thermogravimetric Analysis (TGA) of HNTs, DLHNTs, and DLHNTs-CH

**Figure 3:**
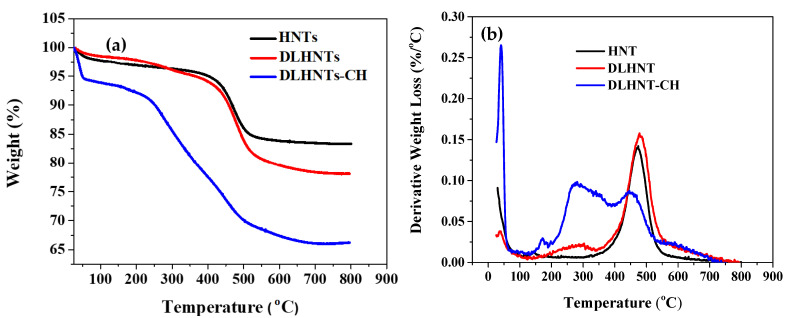
(**a**) TGA plots for HNTs, DLHNTs, and DLHNT-CH; (**b**) Derivative weight loss vs. temperature for HNTs, DLHNTs, and DLHNT-CH.

TGA was done to affirm the successful loading of curcumin into halloysite (HNTs) and verify coating of DLHNTs with chitosan. Figure 3a indicates the TGA profile of the HNTs, DLHNTs, and DLHNTs-CH. Beginning with the decomposition of adsorbed water molecules on the surface of the three materials, all the graphs exhibit a comparable initial stage decomposition at 60 °C. For both HNTs and DLHNTs, it can be observed that there is an initial 2 wt.% loss. However, 6 wt.% loss was observed for the DLHNTs-CH from 25 to 60 °C. This can be ascribed to bound water on the surface of the materials. From 450–500 °C, 6 and 11 wt.% loss is observed for HNTs and DLHNTs, respectively, and can be attributed to the dehydroxylation of structural aluminol groups existing in HNTs [40]. For the DLHNTs, the small shoulder observed around 470–530 °C represents a 6 wt.% loss, which can be due to the thermal decomposition of organic curcumin. However, from 250–500 °C, a 21 wt.% loss is recorded for the DLHNTs-CH. This can be ascribed to the predominant thermal decomposition of chitosan polyelectrolyte coated onto the DLHNTs [41]. Therefore, subjecting the samples to a temperature range of 25–800 °C yielded ca. 17, 22, and 34 wt.% overall weight loss for the HNTs, DLHNTs, and DLHNTs-CH, respectively. From Figure 3b, it can be seen that for HNTs, the main weight loss occurred at ca. 463 °C. However, the DLHNTs recorded significant weight losses at ca. 283 and 464 °C. When the DLHNT was coated with chitosan, the main weight losses were observed at ca. 42, 282, and 463 °C. These weight losses are summarized in Table 2.

### 3.5. Fourier Transform Infrared Spectroscopy (FTIR)

**Figure 4:**
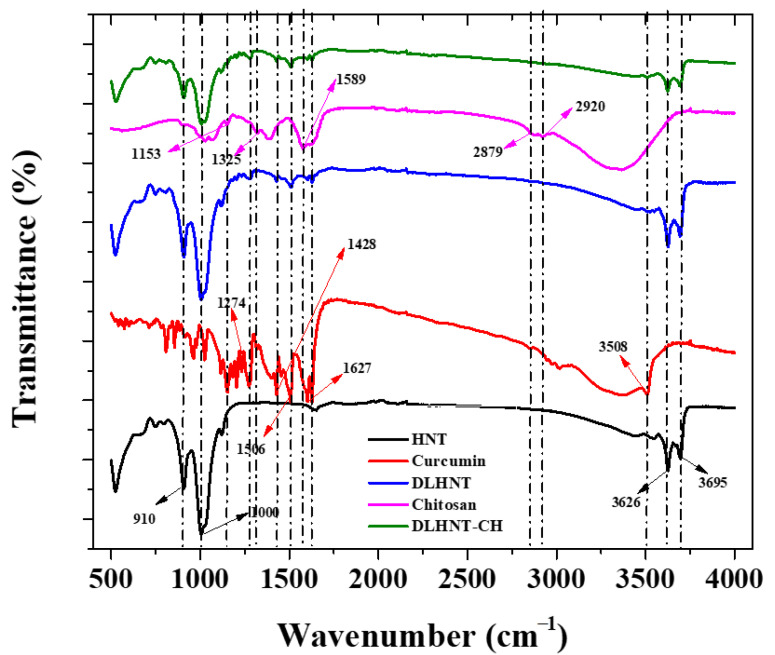
FTIR spectra of HNT, curcumin, DLHNTs, chitosan, and DLHNT-CH.

Figure 4 illustrates the FTIR spectra with all functional groups present in the halloysite (HNTs), DLHNTs, and DLHNTs-CH. The stretching vibrations of the inner surface hydroxyl groups of the HNTs have characteristic peaks at 3695 and 3626 cm^−1^ [42]. The peaks at 910 and 1000 cm^−1^ represent Al–OH bending and the Si–O–Si symmetric stretching [35]. Following the loading of curcumin, the FTIR spectra revealed traditional curcumin bands at 3508 cm^−1^ (attributed to phenolic O–H vibrations), 1627 cm^−1^ (attributed to C=C vibrations of aromatic groups), and 1506 cm^−1^ (attributed to C=O vibrations), 1428 cm^−1^ (attributed to C–H bending vibrations), and 1274 cm^−1^ (attributed to C–O stretching vibrations) [43], indicating the successful loading of curcumin in the HNTs.

The FTIR for chitosan recorded characteristic peaks at 2920 and 2879 cm^−1^ (attributed to asymmetric and symmetric stretching of C–H group), 1589 cm^−1^ (attributed to N-H bending of the primary amine group), 1325 cm^−1^ (attributed to C–N stretching of amide III), and 1153 cm^−1^ (attributed to the asymmetric stretching of C–O–C group) [44]. After coating the DLHNTs with chitosan, the chitosan peaks were not observed. It is likely that the chitosan peaks were overshadowed by the intense peaks of the functional groups present in HNTs and curcumin. The FTIR and TGA results showed that DLHNTs were coated with chitosan.

### 3.6. Scanning Electron Microscopy (SEM) Analysis

**Figure 5:**
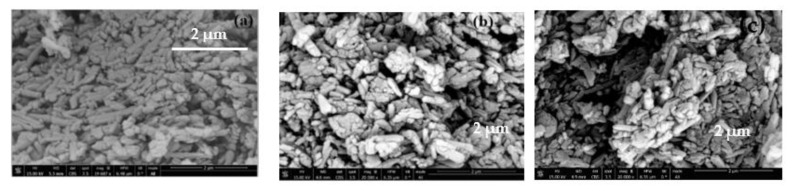
Scanning Electron Microscopy images of (**a**) HNTs, (**b**) DLHNTs, (**c**) DLHNTs-CH.

To study the morphology of halloysite nanotubes (HNTs), DLHNTs, and DLHNTs-CH, scanning electron microscopy analysis was carried out. From Figure 5a, distinct cylindrical tubes consisting of comparable lengths and diameters can be observed. This falls in line with reported morphology of HNTs [28]. The cylindrical and tubular morphology of the nanotubes were maintained after curcumin was loaded into the HNTs (Figure 5b). However, agglomeration of the nanotubes was observed, and this can be due to the drug-loading procedure employed in this work [28]. The agglomeration of the DLHNTs is anticipated to influence the release rate of curcumin in and around the nanotubes. The drugs seen on the surface of the tubes will cause an initial eruption of curcumin in the release medium before sustained release may be achieved by the gradual diffusion of the in-bound curcumin in the lumen of the nanotubes [9]. With the DLHNTs-CH, the further clustering of the nanotubes can be seen in Figure 5c. In spite of the high agglomeration triggered via the coating of the polycation, the halloysite nanotubes still maintained their cylindrical morphology (Figure 5c).

### 3.7. Transmission Electron Microscope (TEM)

**Figure 6:**
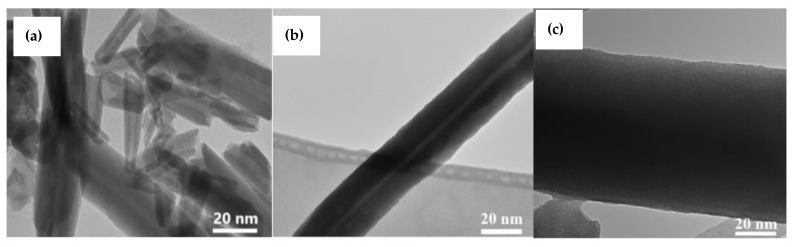
Transmission electron microscopy images of (**a**) HNTs; (**b**) DLHNTs; and (**c**) DLHNTs-CH.

As indicated in Figure 6, TEM images revealed that HNTs are hollow tubular nanomaterials. In comparison to HNTs, the DLHNTs had narrower internal diameters, indicating the presence of drugs in the lumen. This is in line with the results obtained from the nitrogen porosimetry analysis. Again, from Figure 6A, it can be seen that the electron density for the HNTs was lighter but revealed the tubular nature of halloysite. Contrary to this, in Figure 6B a rather dense electron density was observed for the DLHNTs, signifying that curcumin has been loaded into the lumen of HNTs. However, a thin light electron density was observed for these DLHNTs. With the DLHNTs-CH (Figure 6C), a uniform dense electron density was seen throughout the tube; this confirmed the presence of chitosan around the nanotubes.

### 3.8. In Vitro Curcumin Release

**Figure 7:**
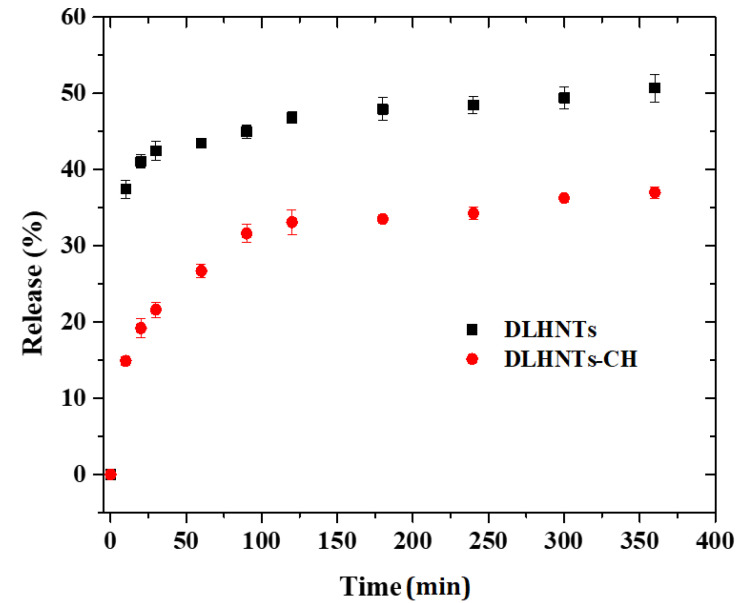
Release profiles of curcumin from DLHNTs and DLHNTs-CH. Experiment was conducted in triplicate.

The release profiles of curcumin from DLHNTs and DLHNTs-CH are shown in Figure 7. From Figure 7, 50.7 and 37% of the curcumin was released, respectively, from DLHNTs and DLHNTs-CH. Evidently, the amount of curcumin released from DLHNTs-CH was lower when compared to that of DLHNTs. The percentage release values reported were achieved within 6 h and expected to increase if the release time is extended. A complete release is expected with a sufficiently longer period of time. Considering Fick’s first law of diffusion, the diffusion rate is proportional to the concentration gradient. The higher amount of curcumin released in the initial stages can be attributed to the large concentration gradient. The slow release profile observed for the DLHNTs-CH could be attributed to the increased diffusion path of the curcumin from the lumen of the HNTs and subsequently from the biopolymer (chitosan) coated on the DLHNT. Thus, it is concluded that sustained release was observed when curcumin was loaded into HNTs; however, the release of curcumin could be controlled when coated with chitosan. In 2013, Jing and colleagues reported similar observations [45]. They stated that most drug release from halloysite nanotubes is preceded by an initial burst release. However, in this work, the effect of the initial burst release was curtailed via the chitosan coating on the surface of the DLHNTs.

### 3.9. Curcumin Release Kinetic Profiles

**Figure 8:**
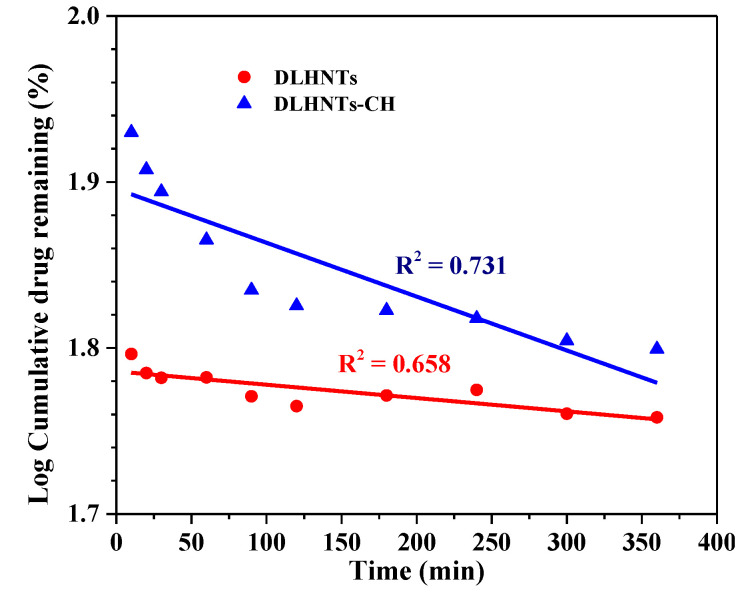
First-order release profile of curcumin from DLHNTs and DLHNTs-CH.

**Figure 9:**
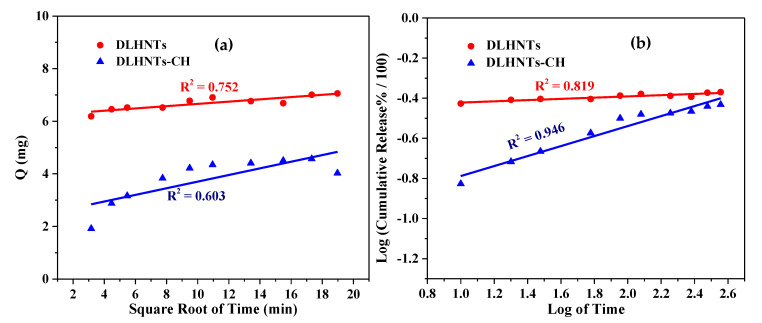
(**a**) Higuchi release profile of curcumin from DLHNTs and DLHNTs-CH; (**b**) the Korsmeyer–Peppas release profile for DLHNTs and DLHNTs-CH.

**Figure 10:**
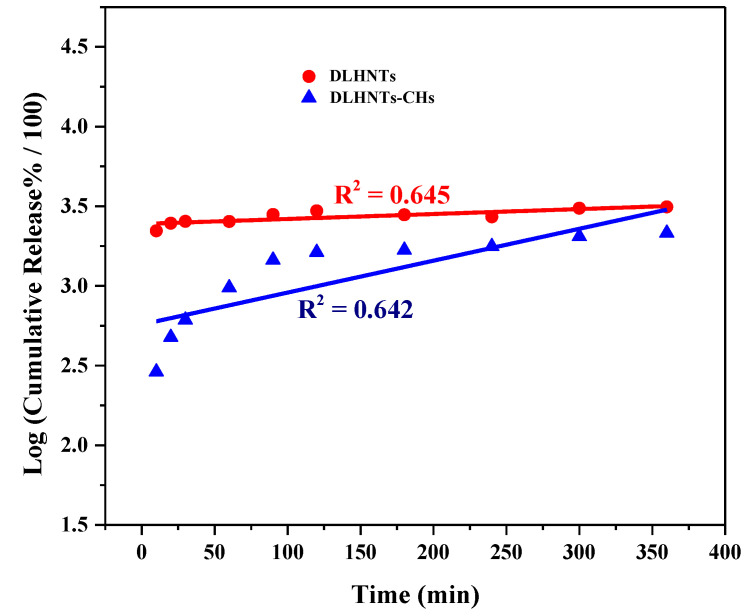
Hixson–Crowell square root release profile of curcumin from DLHNTs and DLHNTs-CH.

Zero-order, first-order, Higuchi, Hixon-Crowell square-root, and Korsmeyer-Peppas kinetic models were used as fitting models for the results that were acquired from the drug release studies. Correlation coefficients were achieved using regression evaluation. ‘Q’ is the amount of curcumin released at time ‘t’ for each plot. Figure 8, Figure 9 and Figure 10 below are plots for the various models. Table 3 is a summary of the rate constants acquired from the kinetic models. From Table 3 and Figure 8, Figure 9 and Figure 10, it can be observed that, with the curcumin-loaded halloysite, a higher correlation was recorded with the Korsmeyer-Peppas model (R^2^ = 0.819) compared to the Higuchi (R^2^ = 0.752), first-order (R^2^ = 0.658), Hixson–Crowell square root (R^2^ = 0.645), and zero-order (R^2^ = 0.632) models. A higher correlation was displayed with the Korsmeyer–Peppas model (R^2^ = 0.946) than zero-order (R^2^ = 0.752), first-order (R^2^ = 0.731), Hixson–Crowell square root (R^2^ = 0.642), and Higuchi (R^2^ = 0.603) models for DLHNTs-CH.

When the release rate of a drug from a vehicle is constant, the release could be termed a zero–order release [46]. The release profiles for all the samples were best described with the aid of the Korsmeyer-Peppas model. In this model, the drug release is diffusion controlled, and it is characterized by using the value of the release exponent (n). When the release of a drug loaded into a vehicle follows a steady gradual diffusion, it could be classified as Fickian diffusion. Thus, curcumin diffuses out of the DLHNTs and DLHNTs-CH in a controlled and sustained manner. There was a clear relationship between the rate constants and the nature of release from the vehicles. It can be seen that the DLHNTs-CH achieved sustained/controlled release. From Table 3, the values of n were 0.031 and 0.249 for DLHNTs and DLHNTs-CH, respectively. The calculated values of the release exponent (n) are much less than 0.45, signifying that the release follows the Fickian release behavior of drugs from cylindrical particles.

### 3.10. MTT Assay

With human cancer cell lines like K562, HL60, MCF-7, and Hela, curcumin has been shown to inhibit the growth [47]. In this study, the effect of different curcumin doses (0–150 µm) on the MCF-7 cell line purchased from ATCC (Manassas, VA, USA) was studied. After 24 h of cell treatment, curcumin demonstrated high levels of inhibitory effects on the breast cancer cell line MCF-7, with an IC_50_ of ~30 μm.

### 3.11. Trypan Blue Exclusion Assay

**Figure 11:**
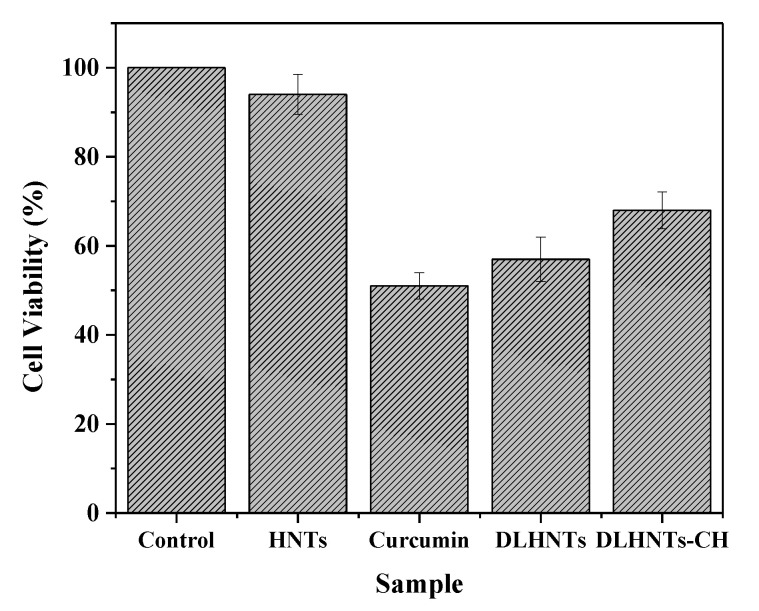
Cell viability assay using Trypan Blue Exclusion Assay. Experiment was conducted in triplicate.

The viability of the drug-treated samples was compared to the untreated MCF-7 cells. From Figure 11, it can be seen that after 24 h of incubation, the HNTs-treated cells recorded cell viability greater than 94%, while 51% cell viability was recorded for the curcumin-treated cells. On the other hand, the wells treated with DLHNTs recorded a cell viability of 57%.

The physiological microenvironment of the cell media triggered curcumin to dissolve very quickly, losing its efficacy in a short period of time (poor bioavailability and distribution). When this happens, the cells that were not affected by the curcumin drug will be able to proliferate within a short period of time, avoiding the effect of the residual drug (the cells tend to develop resistance against the drug). This is comparable to the results obtained from drug release studies, as seen in Figure 7. The wells treated with DLHNTs-CH recorded 68% cell viability. This is due to the sustained/controlled release of lethal concentrations of curcumin over a relatively longer time period. From Figure 7, DLHNTs-CH achieved 37% release of curcumin after 6 h, which continued beyond the 6 h. This could mean that the residual amount of curcumin available to the cells after 24 h of drug release from the DLHNTs-CH was still higher. The unexpected increment of cell visibility from the DLHNTs is probably due to the short time allowed for complete release of the drug from the HNT lumen. The trend is also revealed in the polycation-coated HNTS. In future works, the authors expect to extend the release time using more stable cell lines to be able to corelate the effect of the control release on cell viability.

From the results obtained, we could conclude that polycation chitosan only was not toxic to the MCF-7 cells.

It is sufficient to say that there are numerous works in the literature on the usage of HNTs as drug carriers; however, detailed comparative release kinetic studies both in hypotonic and diseased cells are not well characterized, suggesting a careful study to correlate such studies for future detailed in vivo studies. In this work, we determined the release kinetics of curcumin using a different model equation to ascertain the drug control release characteristics. Further works were conducted to affirm the efficacy of the drug release in diseased cells. It was noted that more stable cells with extended period cell culturing are required to confirm the bust-release vis-a-vis the control release.

## 4. Conclusions

In this work, key parameters were evaluated to affirm the release properties of curcumin-loaded HNTs onto cancer cells. The slow and steady release of the drug from the nanocomposite was determined by correlation coefficients measurements using predictive modeling. The Korsmeyer-Peppas model was found to support the release mechanism of curcumin into cancer cells in vitro. However, it was noticed that the drug-loaded HNTs and DLHNTs-CH failed to demonstrate effective cell death as compared to the raw drug, which is attributed to the shorter time period allowed for most of the drug to be released onto the cells. In future works, the authors will use stable cell lines to correlate control release with cell viability. Thus, the polycationic coated HNTs have the potential to serve as a drug carrier for delivery onto various cancer cells in vitro.

## Figures and Tables

**Table 1:** Tabulated values (bottom) for surface area and pore volume for HNTs and DLHNTs.

Material	Surface Area (m^2^/g)	Pore Volume (cm^3^/g)
HNT	38.95	0.11
DLHNT	18.21	0.04

**Table 2:** Tabulated values (bottom) showing the difference in weight loss (reported at temperatures where the derivative weight loss plot shows peak in Figure 3b of the various samples).

Sample	Temperature (°C)	Weight Loss (%)
HNTs	463	~10
DLHNTs	283	~3.5
	464	~10.8
DLHNTs-CH	42	~3.5
	282	~12.7
	463	~27.4

**Table 3:** Tabulated values (bottom) of rate constants of different kinetic models.

Model	Parameter	DLHNTs	DLHNTs-CH
Zero-Order C = K_o_t	R^2^	0.632	0.752
	K_o_	0.17	0.14
First-Order Log C_o_ − Log C_t_ = K_1_t/2.303	R^2^	0.658	0.731
	K_1_	−8.03 × 10^−5^	−3.24 × 10^−4^
Higuchi Log Q = Log K_H_ + 1/2 Log t	R^2^	0.752	0.603
	K_H_	6.23	2.44
Korsmeyer-Peppas Log (Mt) = Log K_kp_ + n Log t	R^2^	0.819	0.946
	K_kp_	−0.45	−1.04
	n	0.031	0.249
Hixson–Crowell model Q_0_^1/3^ − Q_t_^1/3^ = K_HC_t	R^2^	0.645	0.642
	K_HC_	3.11× 10^−4^	0.002

## Data Availability

The data presented in this study are available on request from the corresponding author. The data are not publicly available due to the fact that this work is part of an ongoing research.
